# Exploring the Prognosis-Related Genetic Variation in Gastric Cancer Based on mGWAS

**DOI:** 10.3390/ijms242015259

**Published:** 2023-10-17

**Authors:** Yuling Zhang, Yanping Lyu, Liangping Chen, Kang Cao, Jingwen Chen, Chenzhou He, Xuejie Lyu, Yu Jiang, Jianjun Xiang, Baoying Liu, Chuancheng Wu

**Affiliations:** 1 Department of Preventive Medicine, School of Public Health, Fujian Medical University, Fuzhou 350108, China; zyl120821@fjmu.edu.cn (Y.Z.); yanyang@fjmu.edu.cn (Y.L.); clp2220220200@fjmu.edu.cn (L.C.); flechazo@fjmu.edu.cn (K.C.); cjw-july@fjmu.edu.cn (J.C.); hcz@fjmu.edu.cn (C.H.); lvxuejie@fjmu.edu.cn (X.L.); jiangyu@fjmu.edu.cn (Y.J.); jianjun.xiang@fjmu.edu.cn (J.X.); lby@mail.fjmu.edu.cn (B.L.); 2 The Key Laboratory of Environment and Health, School of Public Health, Fujian Medical University, Fuzhou 350108, China

**Keywords:** gastric cancer, plasma metabolites, metabolome genome-wide association studies, prognosis

## Abstract

The use of metabolome genome-wide association studies (mGWAS) has been shown to be effective in identifying functional genes in complex diseases. While mGWAS has been applied to biomedical and pharmaceutical studies, its potential in predicting gastric cancer prognosis has yet to be explored. This study aims to address this gap and provide insights into the genetic basis of GC survival, as well as identify vital regulatory pathways in GC cell progression. Genome-wide association analysis of plasma metabolites related to gastric cancer prognosis was performed based on the Generalized Linear Model (GLM). We used a log-rank test, LASSO regression, multivariate Cox regression, GO enrichment analysis, and the Cytoscape software to visualize the complex regulatory network of genes and metabolites and explored in-depth genetic variation in gastric cancer prognosis based on mGWAS. We found 32 genetic variation loci significantly associated with GC survival-related metabolites, corresponding to seven genes, *VENTX*, *PCDH 7*, *JAKMIP1*, *MIR202HG*, *MIR378D1*, *LINC02472*, and *LINC02310*. Furthermore, this study identified 722 Single nucleotide polymorphism (SNP) sites, suggesting an association with GC prognosis-related metabolites, corresponding to 206 genes. These 206 possible functional genes for gastric cancer prognosis were mainly involved in cellular signaling molecules related to cellular components, which are mainly involved in the growth and development of the body and neurological regulatory functions related to the body. The expression of 23 of these genes was shown to be associated with survival outcome in gastric cancer patients in The Cancer Genome Atlas (TCGA) database. Based on the genome-wide association analysis of prognosis-related metabolites in gastric cancer, we suggest that gastric cancer survival-related genes may influence the proliferation and infiltration of gastric cancer cells, which provides a new idea to resolve the complex regulatory network of gastric cancer prognosis.

## 1. Introduction

Gastric cancer is one of the most burdensome malignancies worldwide, with insidious onset, rapid progression, and poor prognosis. Genetic variants play an important role in the progression of gastric cancer, and familial aggregation exists in approximately 10% of gastric cancer patients [1]. Gastric cancer is also a metabolism-related disease, and gastric cancer reprograms the pathways of nutrient acquisition and metabolism, thereby enhancing the growth and survival of gastric cancer cells [2]. The first two parts of this study have comprehensively explored the deep genetic variation in gastric cancer and its complex metabolic map from gastric cancer genome and metabolomics, providing a genome-wide molecular understanding about gastric cancer and discovering the unique metabolic fingerprint of gastric cancer, which provides clues for targeted therapeutic research of gastric cancer and helps the progress of personalized therapy.

However, genes cannot function in isolation, and the integration of disparate histological datasets at the gene level and at the level of gene networks is a key approach to further improve our systems-level understanding of the genetic basis of cancer. The use of individual techniques of metabolomics also often fails to uncover the complexity of its full biological information, and, with the metabolome degree downstream of genomics, transcriptomics, and proteomics, mapping the complete metabolic changes under specific conditions associated with pathogenic factors, host, or environmental co-effectors, it is more important to combine metabolomics with other histological approaches to gain a more comprehensive understanding of gastric carcinogenesis and development [3]. Studies have shown that genetic variations play a significant role in regulating important metabolic carcinogens in tumors. For instance, Reimann et al. [4] identified potential biomarkers of osteosarcoma, such as the WNT pathway genes, *IGF1/W52,* the *W51R* homodimer signaling pathway genes *W53* and *EI24*, *MUC4*, and other mucin genes, as well as *CDC27*. Additionally, Cao et al. [5] discovered that rs 61991156 in *MIR379* was linked to poor glycolysis in gastric cancer by enhancing the regulation of PKM2, which affects the differentiation of gastric cancer cells and patient survival after surgery. Therefore, integrating the two analyses in cancer research can comprehensively elucidate the genetic basis of cancer prognosis and the molecular alterations associated with gastric cancer prognosis, which is crucial for discovering efficient and reliable prognostic markers and exploring prognostic regulatory mechanisms.

Metabolome Genome-Wide Association Study (mGWAS) is the use of the metabolome as an intermediate bridge between genome and phenotype and can be a powerful tool to localize functional genes for complex diseases. mGWAS can describe which DNA variants have a significant effect on metabolite concentrations and identify genetic influences of metabolic phenotypes (GIM), extending our knowledge of the genetic contribution to human metabolomics and enabling genome-wide association studies with metabolomics [6]. Currently, mGWAS studies have been gradually applied to the molecular mechanisms of diseases and biomedical and pharmaceutical studies [7,8]. Unfortunately, no studies related to the application of mGWAS in the prognosis of gastric cancer have been found.

Therefore, in this study, based on the association analysis of plasma metabolites and gastric cancer prognosis, a genome-wide association analysis of 24 plasma metabolites associated with gastric cancer prognosis was performed. In addition, GO enrichment analysis was performed on the possible functional genes identified to explore the functions of metabolism-related genes in gastric cancer prognosis and their biological mechanisms and pathways involved. After that, the correlation between functional gene expression and survival outcome of gastric cancer patients was further explored in the TCGA database. Finally, the gene information was mapped in the metabolic network with the help of the Cytoscape software to map the gene–metabolism interactions. The results of this part of the study fill the gap of mGWAS studies in gastric cancer prognosis, provide a new idea to deeply elucidate the genetic basis associated with gastric cancer survival, and indicate important regulatory pathways during gastric cancer cell progression. In addition, the functional genes localized in this study may serve as biomarkers for gastric cancer prognosis, which may also provide clues for the development of new targeted drugs and promote the progress of personalized therapy, which is of great significance for improving the quality of patient survival and reducing the survival rate of patients.

## 2. Results

### 2.1. Basic Characteristics of Research Subjects

A total of 251 patients were included in this study, according to the exclusion and inclusion criteria, including 33 cases due to difficulties in investigation due to family moving out of town, refusal to investigate, and unreachability, with a follow-up rate of 86.85%, and, finally, a total of 218 patients were included in the follow-up analysis. Among them, 162 cases were male, 56 cases were female, and the survival rates of the total population at 1, 3, and 5 years were 61.5%, 40.8%, and 37.5%, respectively, with a median survival period of 22.0 months. As shown in Table 1, patients’ ages, TNM stages, surgeries, and chemotherapies were correlated with their prognoses (*p* < 0.05). Among them, ages greater than or equal to 65 years or intermediate to advanced TNM stage were the high-risk factors for poor prognosis, whereas surgery and chemotherapy decreased the risk of poor prognosis in gastric cancer.

To further investigate the potential correlation between functional genes related to survival and the prognosis of gastric cancer, we obtained gene expression data from Asian patients with gastric adenocarcinoma from The Cancer Genome Atlas (TCGA) database. Our study analyzed the relationship between these survival-related gene expressions and the prognosis of gastric cancer. The study included 58 patients with gastric adenocarcinoma, consisting of 34 males and 24 females, with an average age of (63.8 ± 11.4) years old. The median survival time was 36.0 months, with survival rates of 87.16% and 65.17% for 1 and 3 years, respectively. The patients’ pathological stage included 4 cases of early stage (stage I), 51 cases of middle stage (stage IIA, IIB, and III), and 3 cases of late stage (stage IVA and IVB). These patients were selected from the TCGA database.

### 2.2. Metabolic Profiles of Plasma Samples

**Figure 1:**
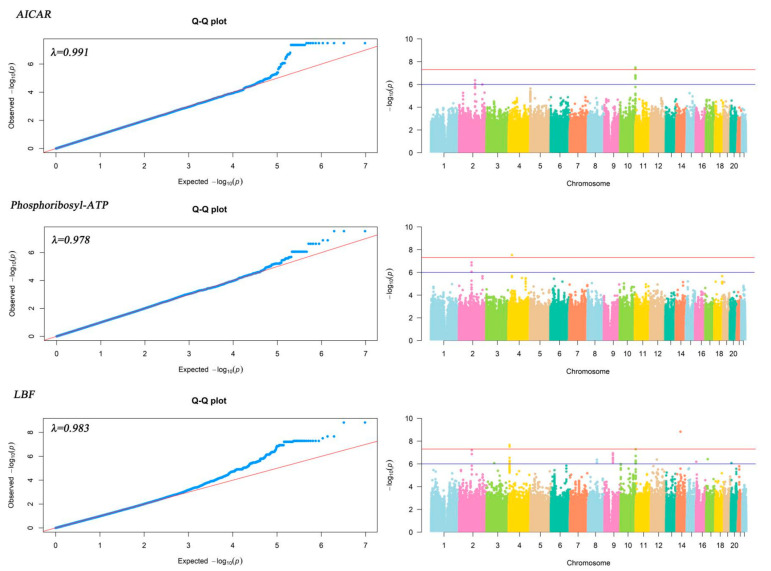
QQ and Manhattan plot of the results of genome-wide association analysis of plasma metabolites associated with gastric cancer prognosis.

In this part of the study, we used generalized linear model (GLM), adjusted for the first three principal components of genome-wide genetic variant loci, age, sex, TNM stage, surgery, chemotherapy, and other confounding factors, and performed the association analysis of 24 prognosis-related plasma metabolites and genome-wide genetic variant loci for gastric cancer, as described above. The results showed that three metabolites were significantly associated with 32 SNP loci (corresponding to seven genes, *p* < 5 × 10^−8^), as shown in Table 2. In addition, there were 772 SNP loci (corresponding to 206 genes) suggestively associated with 23 plasma metabolites (*p* < 1 × 10^−6^). Figure 1 shows the QQ and Manhattan plots of the results of metabolite genome-wide association analysis for the three metabolites. The three QQ plots show that the previous associations between metabolites and single nucleotide polymorphic loci found in this study were the result of natural selection rather than random drift. The inflation factors of genome-wide association analysis for all three metabolites were close to 1, indicating that there was no significant population stratification and fewer false positives in this study. The conclusions were reliable. Manhattan plots visualized the results of the genome-wide association analysis of the three metabolites.

### 2.3. Functional Gene GO Pathway Enrichment Analysis

**Figure 2:**
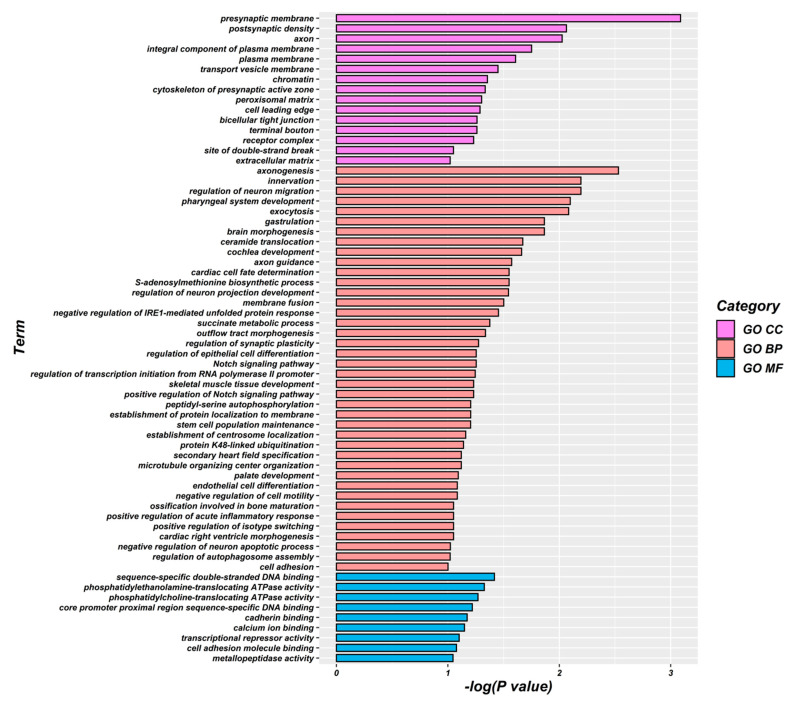
Histogram of GO enrichment results of plasma metabolite-related genes associated with gastric cancer prognosis.

**Figure 3:**
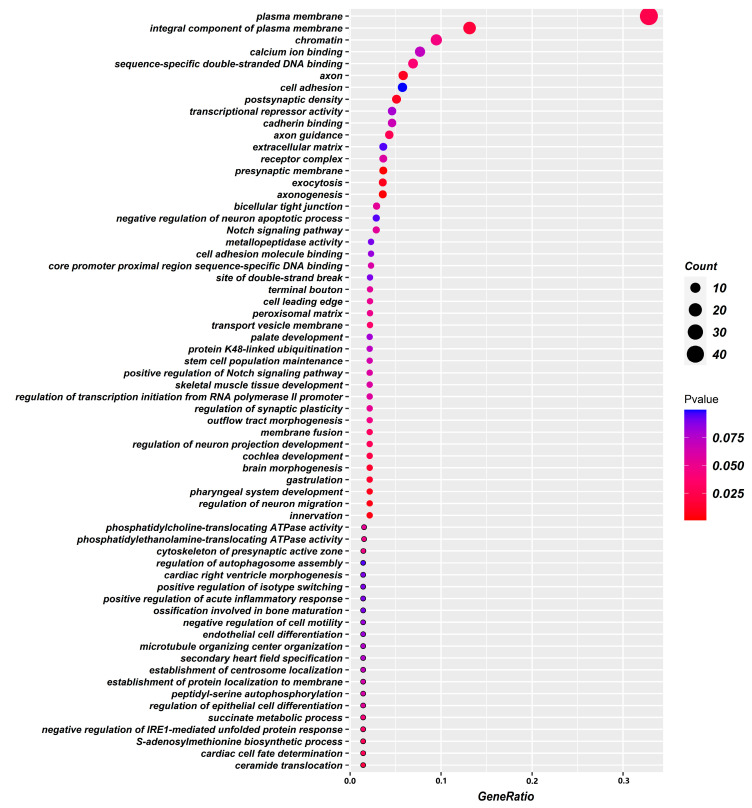
Bubble plot of GO enrichment results of plasma metabolite-related genes associated with gastric cancer prognosis.

To further explore the genetic variation mechanism of gastric cancer survival-associated metabolites and the biological processes involved in their regulation, we analyzed 206 potential genes that were identified through a genome-wide association analysis of these metabolites for GO enrichment. The results showed that gastric cancer survival-related metabolites were mainly associated with the presynaptic membrane, postsynaptic density, axon, plasma membrane, transport vesicle membrane, chromatin, presynaptic active zone cytoskeleton, extracellular matrix, and other cellular fractions and were mainly involved in axonal genesis, innervation, regulation of neuronal migration, pharyngeal system development, cytokinesis, S-adenosylmethionine biosynthesis process, skeletal muscle tissue development, and stem cell population maintenance. The molecular functions include sequence-specific double-stranded DNA binding, phosphatidylethanolamine transporter ATPase activity, phosphatidylcholine transporter ATPase activity, sequence-specific DNA binding in the proximal region of the core promoter, calmodulin binding, calcium ion binding, transcriptional repression activity, and cell adhesion molecule binding. Adhesion molecule binding, etc. (Figure 2 and Figure 3).

### 2.4. Analysis of the Association between Functional Gene Expression and Survival of Gastric Cancer

**Figure 4:**
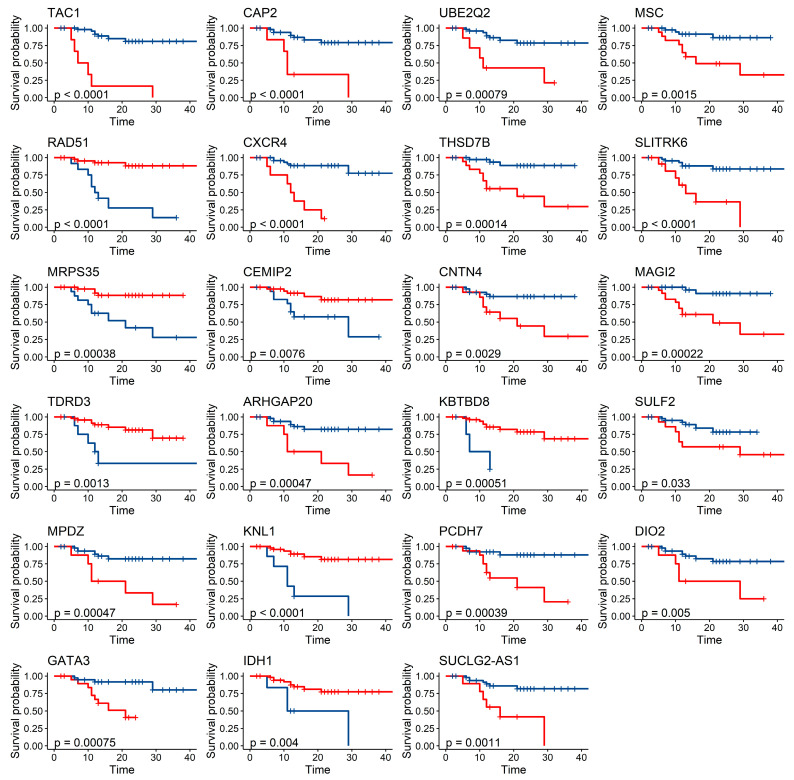
Survival curve of functional genes of plasma metabolite localization associated with gastric cancer prognosis. The unit of time is month.

To further explore the association of possible functional gene expression with gastric cancer prognosis, this study further searched the TCGA database for the 206 gene expression data localized above, and a total of 195 gene expression data were retrieved. A total of 58 patients with Asian gastric adenocarcinoma were included in this part of the TCGA study. After adjusting for age, gender, and TNM stage, multifactorial Cox regression showed that 23 genes were associated with survival in gastric cancer patients, and their survival curves were plotted in Figure 4.

### 2.5. Construction of Metabolite Gene Interaction Networks

**Figure 5:**
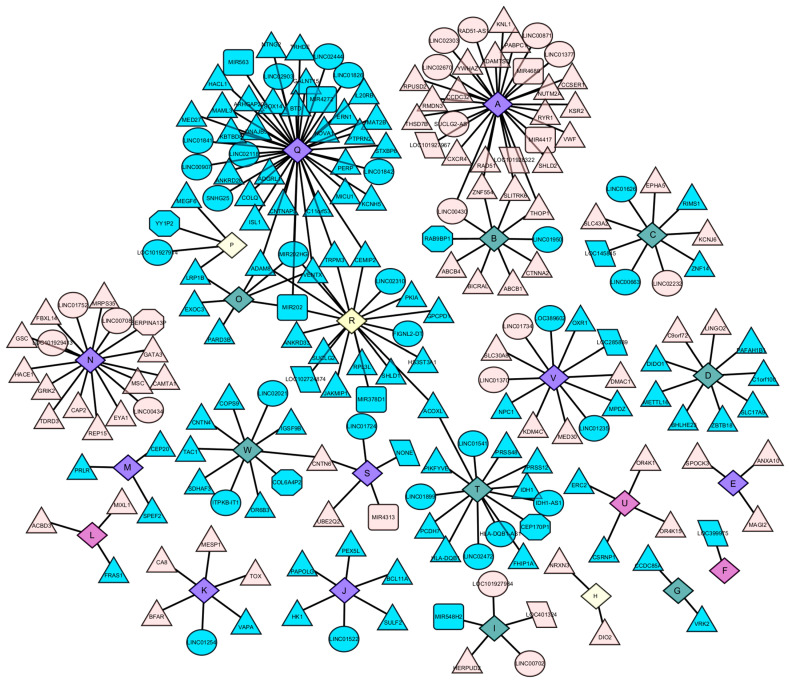
Metabolite-gene interaction network diagram. A: Arachidonic acid; B: Guanosine diphosphate mannose; C: P1,P4-Bis (5′-uridyl) tetraphosphate; D: 5′-Methylthioadenosine; E: Cer (d18:0/12:0); F: D-(+)-Tryptophan; G: Uridine diphosphategalactose; H: Paraxanthine; I: Uric acid; J: Palmitoyl sphingomyelin (SM(d18:1/16:0)); K: Indole-3-lactic acid; L: Ornithine; M: Porphobilinogen; N: Dipalmitoylphosphatidylcholine; O: AICAR; P: Methylacetoacetic acid; Q: DL-Dipalmitoylphosphatidylcholine; R: LBF; S: Linoleic acid; T: Phosphoribosyl-ATP; U: Phenylacetylglutamine; V: Glycoursodeoxycholic acid; W: Inosine triphosphate.

Based on the results of genome-wide association analysis of metabolites, this study visualized the regulatory network map between 23 metabolites and 206 functional genes by the cytoscape software (Figure 5). The central node of each network is the metabolite, and the surrounding nodes are the genes associated with the metabolite. The shapes represent the types of genes, triangles indicate coding genes, circles are lncRNAs, squares are miRNAs, octagons are pseudogenes, and the rest are parallelograms. The color represents the direction of action of genes and metabolites, blue indicates genes negatively regulating metabolites, and red indicates genes positively regulating metabolites. The different types of metabolites are also represented by colors, purple for amino acid metabolites, dark blue for lipid metabolites, dark green for nucleotide metabolites, yellow for peptides, and the rest are light yellow.

## 3. Discussion

In this study, we explored the intrinsic genetic variants in gastric cancer prognosis individuals based on mGWAS and identified 32 genetic variant loci significantly associated with gastric cancer survival-related metabolites, corresponding to 7 genes, namely, *VENTX*, *PCDH7*, *JAKMIP1*, *MIR202HG*, *MIR378D1*, *LINC02472*, and *LINC02310*. In addition, this study also identified 722 SNP loci, suggesting an association with prognosis-related metabolites in gastric cancer was identified, corresponding to 206 genes. These 206 possible functional genes for gastric cancer prognosis were mainly involved in cellular components related to cell signaling molecules, such as cellular synapses, axons, and transport vesicle membranes, which are mainly involved in organism growth and development, and neurological regulatory functions related to gastric cancer. The expression of 23 of these genes was shown to be associated with survival outcome of gastric cancer patients in the TCGA database.

*VENTX* is a homologous cassette proteins associated with the proliferation and differentiation of human hematopoietic and immune cells. It has been found to act as a novel lymphatic enhancer factor/T cell factor (LEF1/TCF)-associated transcriptional repressor and is a putative tumor suppressor in chronic lymphocytic leukemia [9]. Immunosuppression of the tumor microenvironment (TME) is thought to be responsible for cancer evasion of immune destruction and is considered as a potential site of intervention, with tumor-associated macrophages (TAM) being a key component of TME. An ex vivo study found that *VENTX* expression was significantly reduced in TAM of colon cancer patients, which could drive the intracellular signaling pathway TAM toward M1 phenotype, reversing immunosuppression in the tumor microenvironment and regulating TAM plasticity and immune status [10]. In addition, *VENTX* expression has been found to promote phagocytosis and immunity in pancreatic cancer, and *VENTX*-modulated TAM has a strong inhibitory effect on tumorigenesis in vivo [11]. However, its role in gastric cancer is not yet clear and needs to be explored in depth.

Protocadherin(PCDH) is the largest subfamily of cadherin family that strengthens synapse and plays a role in signal transduction [12]. Previous studies have found that protocadherin 7 (*PCDH7*) is aberrantly expressed in several cancers and exerts pro- or anti-tumor effects. In cervical cancer, *PCDH7* expression was associated with lymph node metastasis and cell differentiation, and upregulation of *PCDH7* significantly inhibited the proliferative capacity, migratory potential and invasive ability of cancer cells [13], whereas, in lung cancer, *PCDH7* was used as a risk gene for poor prognosis [14], suggesting that *PCDH7* plays different roles in different cancer types. In gastric cancer, knockdown of *PCDH7* enhances cell migration and invasion [15], and *PCDH7* can act as a tumor suppressor to inhibit cell migration and invasion by inhibiting cadherin [16].

Janus Kinase And Microtubule Interacting Protein 1 (*JAKMIP1*) is mainly expressed in neural tissues and regulates multifaceted roles in neuronal mRNA translation [17] and is also involved in the development of various tumors. An in vitro study found that its over-expression was associated with Wnt/β-catenin pathway activation and promoted cancer cell proliferation in vitro [18], and it could serve as a novel effector memory gene that inhibits T cell-mediated cytotoxicity [19]. In addition, a genomic study on DNA methylation and breast cancer risk found that methylation of *JAKMIP1* was associated with breast cancer risk [20]. *JAKMIP1* was also found to be closely associated with gene expression levels of CD8 in colon cancer [21]. However, its role in gastric cancer has not been identified yet.

Non-coding RNAs are RNAs that do not encode proteins, mainly including lncRNAs and miRNAs, etc., and their roles in gastric carcinogenesis, infiltration, and migration have been widely discussed [22,23]. In this study, mGWAS-based studies localized four non-coding RNAs associated with gastric cancer prognosis, namely, *MIR202HG* (MIR202 host gene), *MIR378D1*, *LINC02472*, and *LINC02310*. *MIR378D* expression is a good prognostic factor for patients with esophageal squamous cell carcinoma and regulates the malignant phenotype of tumor cells through AKT [24]. In addition, miR-378d also affects cancer chemotherapy sensitivity. *MIR378D* can undergo adaptive changes in low-dose ionizing radiation-induced non-small cell lung cancer A549 cell lines [25], and chemotherapy-triggered exosomes *MIR378DA-3P* and *MIR378D* can promote breast cancer stemness and chemoresistance [26]. *LINC02310* has hardly been reported. Only a few scholars found that its overexpression would lead to poor prognosis in lung adenocarcinoma patients [27], CCK-8 assay and colony formation assay also showed that it could act as an enhancer in lung adenocarcinoma [28], and downregulation of *LINC02310* could prevent proliferation, migration, invasion, and the epithelial–mesenchymal transition of non-small cell lung cancer cells, promote apoptosis, and enhance radiosensitivity. However, the role of these non-coding RNAs in the prognosis of gastric cancer is not yet clear and needs to be further mapped in the future.

In addition, this part of the study also identified 206 possible functional genes for gastric cancer prognosis, and the TCGA database further argues the credibility of the results of this study. GO enrichment analysis can reveal three functional information about the biological processes involved, the cellular location, and the molecular functions performed by the genes and has become a powerful tool for extracting and interpreting the potential biological mechanisms of high-throughput molecular measurements. After GO enrichment analysis, it is suggested that these functional genes are mainly closely related to cell signaling molecular transmission, organism growth and development, nervous system regulation, and immune escape-related pathways. This study is based on our previous research [29,30,31], which screened gastric cancer survival-related metabolites mainly involved in linoleic acid metabolism, purine metabolism. Linoleic acid (LA) (n-6) is one of the essential fatty acids. n-6 unsaturated fatty acids are similar to cytokine signaling and inflammasome formation in the body and are mainly considered as pro-inflammatory components of the innate immune response [32]. Uncontrolled and inappropriately activated acute inflammation due to excessive inflammatory stimulation provides an ideal tumor microenvironment. Persistent inflammation is associated with cancer risk and metastaticity [33,34]. Upregulation of immune pathways in cancer is associated with poor prognostic subtypes, which may be induced by accumulated unsaturated fatty acids that interact with multiple immunomodulators [35]. In addition, studies have found that linoleic acid metabolites modulate neuroinflammatory responses in advanced Alzheimer’s disease to ameliorate neurodegenerative lesions [36]. Disturbances in linoleic acid metabolism are also seen in peripheral neuropathy induced by the cancer chemotherapeutic drug paclitaxel [37]. Purines are the basic components of nucleotides, which are one of the hallmarks of infinitely proliferating cancer cells. Purines also provide the necessary energy and cofactors to promote cell survival and proliferation, and their presence in the body is mainly in the form of purine nucleotides (adenosine). Adenosine-based cellular communication systems are present in almost all components of the body, and adenosine is one of the most pleiotropic biochemical components of the tumor microenvironment that affects host and tumor responses. Adenosine exerts potent immunosuppressive and anti-inflammatory activities in the body, and currently targeted purine drugs are used in the treatment of clinical cancers [2,38]. In addition, adenosine may function as a neurotransmitter, and purine signaling plays a role in neurological related diseases [39,40].

The intestinal nervous system (ENS) is the largest component of the peripheral nervous system and is very similar to the components and functions of the central nervous system. It is a key regulator of intestinal barrier function and a regulator of intestinal homeostasis. The intestinal nervous system is a network of intestinal neurons and glial cells, which originates from neural crest stem cells. The neural callus stem cells originate from neuroectoderm and undergo the processes of proliferation, migration, and differentiation, with the participation of various cytokines and signaling molecules, forming various types of intestinal cells. These cells express different neurotransmitters and neuropeptides, respectively, which jointly regulate intestinal function. BeateNiesler et al. [41] summarized the role of ENS in gastrointestinal and systemic diseases and highlighted the interaction of ENS with key factors affecting disease phenotyping. These functional genes may play a similar role in the nervous system and gastrointestinal system because they are all composed of the same cell types and molecular mechanisms. Therefore, combined with the results of enrichment of functional genes and metabolites, this study predicts that these functional genes mainly mediate the regulation of metabolites through related pathways, such as cell signal molecule transmission, body growth and development, nervous system regulation, and immune escape, and then affect the prognosis of gastric cancer.

## 4. Materials and Methods

### 4.1. Study Population and Sample

Patients with gastric cancer were derived from new cases at Xianyou County Affiliated Hospital, Fujian Province, China. The inclusion criteria for patients were (1) surgically or endoscopically obtained tissue samples, pathologically new cases; (2) confirmation date from April 2013 to November 2017; and (3) living in Xianyou for more than 10 years. We also used the following exclusion criteria: (1) identified known congenital diseases; (2) suffering from metabolic diseases, severe heart, lung, liver, kidney, and neurological and psychiatric diseases; (3) women who were pregnant, breastfeeding, or could not exclude the possibility of pregnancy; (4) alcoholics, drug addicts, and long-term users of proton pump inhibitors, hormones, or non-steroidal anti-inflammatory drugs; (5) chronic inflammatory diseases; (6) within the last 2 weeks, any acute medical manifestation; (7) those with major stress reactions (e.g., psychological trauma, burns) within the last 2 weeks; and (8) those with hematological disorders, including various leukemias, various anemias, etc.

In this study, a prospective case follow-up study design was used to obtain complete survival information and clinical data through annual data by extracting all causes of death, case data, and follow-up data conducted by village doctors. Finally, a total of 218 individuals were included in this study for follow-up analysis.

Five milliliters of fasting peripheral venous blood were collected from patients. Blood samples were placed in EDTA anticoagulated tubes; centrifuged at 3000× *g* for 10 min; then loaded into plasma, white blood cells, and red blood cells; and stored in a −80 °C refrigerator. All subjects consented to inclusion in the study prior to participation. All procedures involving human participants were performed in accordance with institutional and National Research Council ethical standards and the 1964 Declaration of Helsinki and its subsequent amendments or similar ethical standards.

This study was approved by the Bioethics Committee of Fujian Medical University (Fu Medical Ethics Review No. 97).

### 4.2. Peripheral Blood Genotyping

In this study, we conducted whole-genome genotyping using The Precision Medical Research Array from Axiom™ (Thermo Fisher Scientific, Waltham, MA, USA) to comprehensively investigate genetic variations associated with gastric cancer prognosis. The PMRA chip, which was launched by Affimatrix in 2016, is specifically designed for precision medicine and contains approximately 900,000 SNP probes, with SNP markers covering the entire genome on average. We then used the Cox proportional risk regression model to explore SNP loci related to gastric cancer prognosis and to identify potential functional genes. We further investigated the biological functions of survival-related genes through GO enrichment analysis. Finally, we examined the correlation between the expression of functional genes identified in this study and gastric cancer prognosis in the TCGA database.

### 4.3. Non-Targeted Plasma Metabolite Assays

XCMS software (https://xcmsonline.scripps.edu, accessed on 8 June 2021), was used for data acquisition and processing. Agilent MassHunter software (version B.01.00, Agilent Technologies, Santa Clara, CA, USA) was used for data conversion, and XCMS Online (https://xcmsonline.scripps.edu, accessed on 8 June 2021) was used for data pre-processing, such as peak identification, peak alignment, peak matching, and retention time correction. Secondary mass spectrometry MRM full scan was performed using Agilent quadrupole (Agilent Technologies, Santa Clara, CA, USA) time-of-flight mass spectrometry (q-TOF), and the data were analyzed by combining mass-to-charge ratio (error ± 1 Da), retention time (error ± 0.5 min), and ion patterns in the public human metabolome database (HMDB) [42] and the commercial database from Beijing Boao Company (Thermo mzCloud, accessed on 10 November 2021) for metabolite structure identification and annotation.

### 4.4. Quality Control

Quality control was carried out according to the following criteria: (1) using DQC (Dish Quality Control, calculated from the signal values of several thousand non-polymorphic probes, was used to assess the difference between the signal value distribution and the background signal value, with larger differences indicating better overall experimental procedure and higher typing quality for that sample), but samples with DQC < 0.82 were excluded; (2) genotypes were detected and interpreted using a blinded method, with the detector and the readers did not know whether the samples originated from the gastric cancer group or the healthy group; (3) the typing success rate (call rate) was calculated for each sample (SNP), i.e., [SNPs typed successfully for each sample (SNP)]/[total number of SNPs contained in the microarray (number of all samples undergoing microarray testing)], and samples with call rate < 95% were excluded; (4) X-chromosome inbreeding (pure heterozygous) was estimated for sex, removing samples with ambiguous sex; (5) missing quality control: an excessive number of missing tends to represent lower quality. In this study, 20% was used as the criterion to remove SNPs and samples with deletion rates above 20%; (6) Minor Allele Frequency (MAF) filtering: SNP loci with too low MAF contributed less information and greatly increased the computational effort, leading to an increased probability of false positives, so SNPs with MAF < 0.05 were excluded from this study; (7) The Hardy–Weinberg equilibrium (HWE) test is the most important test in population genetics. In a randomly mated population of infinite size without mutation, migration, and selection, gene frequencies and genotype frequencies will remain constant from generation to generation, and SNP loci with *p* values < 0.05 for the HWE test were excluded from this study; and the (8) Heterozygosity test. In general, in the natural population, the heterozygosity of genotypic individuals is too high or too low, which is not normal, and the deviation may indicate that the sample is contaminated and inbred. In this study, samples with heterozygosity beyond three times the standard deviation were excluded.

After the quality control described above, a total of 218 samples with 254,888 SNP loci remained. Genotype interpolation is a key component of genetic association studies, and this study used the Michigan Imputation Server website [43] (a web-based interpolation service webpage developed by the University of Michigan, USA, which provides a free genotype interpolation service using Minimac4. This webpage proposes a new algorithm for genotype interpolation that improves computational efficiency without loss of accuracy by exploiting local similarities between sequenced haplotypes) to populate genotypes, the populated SNP loci were still quality controlled according to MAF and HWE, and 483,6337 loci were finally retained for analysis.

### 4.5. Data Analysis

In this study, we used Plink software (v1.90 b6.1864, 16 June 2020) for genome-wide association analysis of plasma metabolites associated with gastric cancer and performed genome-wide association analysis of metabolites based on generalized linear models (GLM) based on control of age, sex, TNM stage, etc. The formula *p* = 5 × 10^−8^ was used as the significance threshold for metabolite whole-genome association analysis, and *p* = 1 × 10^−6^ was used as the suggested threshold for metabolite whole-genome association analysis. The QQ and Manhattan maps were plotted using the R [44] software package “qqman” [45] to visualize the results of metabolite-wide genomic association analysis.

And, to further explore the complex regulatory mechanisms of genes and metabolites in the development of gastric cancer, this study performed GO enrichment analysis of all possible functional genes suggested by genome-wide association analysis of metabolites and further verified the correlation between their gene expression and survival of gastric cancer patients in the publicly available database TCGA. Finally, the Cytoscape software was used to visualize the complex regulatory network of genes and metabolites [46].

## 5. Conclusions

In this study, genome-wide association analysis based on prognosis-related metabolites in gastric cancer identified 3 metabolites significantly associated with 32 SNP loci (corresponding to 7 functional genes) and 23 metabolites suggestively associated with 722 SNP loci (corresponding to 206 genes). These functional genes are mainly involved in cell signaling molecular transduction, organism growth and development, nervous system regulation, immune system regulation, and other related pathways, suggesting that gastric cancer survival-related genes may mediate metabolites through these pathways and thus affect the proliferation and infiltration of gastric cancer cells. The results of this part of the study provide a new way of thinking to analyze the complex regulatory network of gastric cancer prognosis, which is beneficial to obtain more prognostic biomarkers and develop new generation of anti-cancer drugs.

## Figures and Tables

**Table 1:** Basic information about the research subjects.

		N	Kaplan–Meier		Cox Regression	
			5-Year of Survival Rate (%)	Log-Rank *p*	HR (95% CI)	*p*
Gender				0.617		
	male	162	37.49		1.00	
	female	56	37.50		0.906 (0.617–1.33)	0.615
Age				0.043 *		
	<65	45	51.11		1.00	
	≥65	173	33.97		1.588 (1.007–2.506)	0.047 *
TNM staging				<0.001 ***		
	early	41	87.80		1.00	
	middle	74	53.40		4.223 (1.771–10.072)	0.001 **
	late	103	5.83		24.457 (10.481–57.069)	<0.001 ***
Surgery				<0.001 ***		
	no	78	16.67		1.00	
	yes	140	49.06		0.345 (0.245–0.484)	<0.001 ***
Chemotherapy				0.007 **		
	no	102	30.39		1.00	
	yes	116	43.76		0.634 (0.454–0.885)	0.007 **
Radiotherapy				0.420		
	no	189	36.34		1.00	
	yes	29	44.83		0.809 (0.48–1.364)	0.427
Tumor location				0.184		
	Not in Cardiac	71	46.48		1.00	
	In Cardiac	147	33.10		1.287 (0.89–1.861)	0.179

HR, hazard ratio; CI, confidence interval; * *p* < 0.05, ** *p* < 0.01, *** *p* < 0.001.

**Table 2:** Genome-wide association analysis results of plasma metabolites associated with Gastric cancer prognosis.

Metabolites	dbSNP *	Chromosome	Alt	Ref	*p*-Value	Gene.refGene
AICAR	rs7076167	10	T	C	3.26 × 10^−8^	*VENTX*
	rs6537595	10	A	T	3.26 × 10^−8^	*VENTX*; *MIR202HG*
	rs4838708	10	A	G	4.40 × 10^−8^	*VENTX*; *MIR202HG*
Phosphoribosyl-ATP	rs11947372	4	T	A	2.93 × 10^−8^	*LINC02472*; *PCDH7*
	rs73107710	4	T	G	2.93 × 10^−8^	*LINC02472*; *PCDH7*
	rs4346608	4	C	T	2.93 × 10^−8^	*LINC02472*; *PCDH7*
LBF	rs7682973	4	A	G	2.17 × 10^−8^	*MIR378D1*; *JAKMIP1*
	rs7157739	14	G	A	1.49 × 10^−9^	*LINC02310*
	rs7158729	14	T	G	1.49 × 10^−9^	*LINC02310*

* For each metabolite we listed only the top three SNP sites.

## Data Availability

The data presented in this study are available on request from the corresponding author. The data are not publicly available due to restrictions privacy.
